# Understanding Developmental Coordination Disorder in Youth Functioning: Measurement Properties of the Spanish Adolescents and Adults Coordination Questionnaire

**DOI:** 10.3390/children12111534

**Published:** 2025-11-13

**Authors:** Laura Delgado-Lobete, Rebeca Montes-Montes, Nerea Blanco-Martínez, Rocío Carballo-Afonso, Carlos Ayán-Pérez

**Affiliations:** 1 Departamento de Atención Sociosanitaria, Facultad de Ciencias Sociosanitarias, Universidad de Murcia, 30800 Lorca, Spain; lauradelgado@um.es (L.D.-L.); rebeca.montes@um.es (R.M.-M.); 2 Departamento de Didácticas Especiáis, Universidade de Vigo, 36310 Vigo, Spain; rociocarballo@uvigo.gal (R.C.-A.); cayan@uvigo.es (C.A.-P.); 3 Well-Move Research Group, Galicia Sur Health Research Institute (IIS Galicia Sur), SERGAS-UVIGO, 36310 Vigo, Spain

**Keywords:** developmental coordination disorder, dyspraxia, adolescence, adults, COSMIN, measurement properties, psychometric validation, questionnaire, assessment, AAC-Q

## Abstract

**Highlights:**

**What are the main findings?**

- This study provides the first comprehensive psychometric evaluation of the AAC-Q-ES, a brief self-report tool assessing daily functional difficulties related to Criterion B of DCD.
- The AAC-Q-ES demonstrated a robust three-factor structure, strong internal consistency, excellent test–retest reliability, and high criterion and construct validity, supporting its conceptual and statistical soundness for use in Spanish adolescents and young adults.

**What are the implications of the main findings?**

- The use of validated instruments like the AAC-Q-ES enables precise detection of functional difficulties, promoting earlier recognition and tailored interventions for DCD in adolescents and young adults.
- The establishment of age- and sex-specific normative percentile bands provides clinicians and researchers with a practical framework to guide diagnosis, monitor progress, and facilitate the assessment of DCD.

**Abstract:**

**Background/Objectives**: The Adolescent and Adult Coordination Questionnaire (AAC-Q) is a brief self-report tool developed to assess daily performance difficulties related to Criterion B of Developmental Coordination Disorder (DCD) across adolescence and adulthood. Despite the AAC-Q’s clinical and research relevance, its psychometric properties have not yet been comprehensively evaluated. This study aimed to examine the structural, construct, and criterion validity, internal consistency, and test–retest reliability of the AAC-Q and to establish normative percentile bands for Spanish adolescents and young adults (AAC-Q-ES). **Methods**: A cross-sectional psychometric study was conducted in 800 typically developing participants (200 adolescents, 600 young adults). Measurement properties—including factor structure, internal consistency, test–retest reliability, construct validity, and criterion validity—were assessed following COSMIN guidelines. Percentile cut-offs were calculated for adolescents and young adults. **Results:** The AAC-Q-ES revealed a three-factor structure with excellent fit indices (CFI = 0.95–0.98; RMSEA = 0.060–0.067). Internal consistency was good (α = 0.76–0.83), and test–retest reliability was excellent (ICC = 0.90, *p* < 0.001). Criterion validity with the Adult DCD/Dyspraxia Checklist was very high in adults (r = 0.972, *p* < 0.001), and construct validity in adolescents was confirmed through moderate correlations with the Flamingo Balance Test (r = −0.352, *p* < 0.01). Age- and sex-specific percentile bands were established. **Conclusions:** The AAC-Q-ES is a psychometrically robust, culturally adapted, and efficient tool for assessing functional difficulties related to DCD in Spanish adolescents and young adults, facilitating both clinical and research applications.

## 1. Introduction

Developmental Coordination Disorder (DCD) is a neurodevelopmental condition that emerges in early childhood and is characterized by persistent difficulties in acquiring and performing motor skills [1]. These difficulties significantly interfere with daily activities and cannot be explained by other medical or psychosocial conditions affecting movement [2,3]. The scientific literature on DCD has mainly focused on its prevalence and clinical manifestations in school-aged children, who represent the most extensively studied population group [2], with a prevalence of approximately 5–6% and a higher incidence in males [1,2,4,5].

Although DCD is now recognized as a chronic condition that persists from childhood into adulthood [6,7], the disorder remains widely underdiagnosed during the early stages of development [6,8]. Furthermore, a considerable proportion of individuals continue to remain unidentified in adolescence and adulthood [2,9]. While several barriers contribute to effective diagnostic services, including long wait lists and poor awareness and understanding of health care professionals and educators regarding DCD, this underdiagnosis is largely attributable to the lack of standardized and age-appropriate diagnostic procedures, which makes it difficult to assess both motor competence and its impact on daily functioning [10]. As a result, many individuals experience long-term difficulties in educational, occupational, social, and recreational participation, along with negative effects on mental health [9], which reinforces the need for early and accurate diagnosis so that appropriate and targeted interventions can be implemented to address these adverse issues.

Diagnosis of DCD is based on four DSM-5 criteria: motor coordination deficits (Criterion A), daily performance limitations and participation restrictions (Criterion B), onset during the developmental period (Criterion C), and exclusion of other explanatory conditions (Criterion D) [1]. However, as highlighted by Armstrong [11], the application of these criteria remains limited, since their verification relies on assessment tools originally developed for children, such as the Movement Assessment Battery for Children-2 (MABC-2) and the Bruininks–Oseretsky Test of Motor Proficiency-2 (BOT-2), which are not suitable for individuals over 16–21 years of age [2,12,13].

To address this limitation, the European Academy of Childhood Disability has emphasized the urgent need to develop diagnostic and screening tools specifically designed for adolescents and adults, capable of accurately capturing the functional impact of DCD on everyday activities and participation [2]. In this context, several self-report instruments have been developed to assess functional limitations related to Criterion B, including the Adult Developmental Co-ordination Disorders/Dyspraxia Checklist (ADC; [14]), the Adolescents and Adults Coordination Questionnaire (AAC-Q; [15]), and the Functional Difficulties Questionnaire-9 (FDQ-9 [16]) for adulthood, with the ADC and AAC-Q being the most widely used in current research [11]. For adolescents, the Adolescent Motor Competence Questionnaire (AMCQ) has been proposed, but its measurement properties have only been partially tested on Australian and Norwegian populations [17,18]. Thus, the number of validated instruments remains limited [2], with the AAC-Q standing out as the only available tool that covers both adolescence and adulthood [15].

The AAC-Q was developed as a brief tool to rapidly identify the risk of DCD in adolescents and young adults through its 12 items, as well as to assess aspects of occupational functioning [15]. It has shown excellent internal consistency, test–retest reliability, and discriminant validity [15,19]. However, only one cross-cultural adaptation has been conducted to date—the European Spanish version [19]. This adaptation, however, did not include an examination of the instrument’s measurement properties, which remain to be established in the Spanish population [19].

Therefore, it is necessary to conduct a comprehensive examination of the measurement properties of the AAC-Q to confirm its reliability and validity in the Spanish adolescent and young adult population. To this end, it is essential to follow guidelines such as those proposed by the COnsensus-based Standards for the selection of health Measurement INstruments (COSMIN) initiative, which establish consensus-based methodological standards for the evaluation of measurement properties, thereby ensuring methodological rigor and a robust psychometric assessment [20]. In this way, it will be possible to generate strong evidence to enhance the clinical and research applicability of the AAC-Q as a brief, effective, and culturally appropriate tool for assessing DCD in adolescents and young adults. Accordingly, the aims of this study were (a) to evaluate the structural validity, construct validity (via hypothesis testing), criterion validity, internal consistency, and test–retest reliability of the AAC-Q adapted to European Spanish, and (b) to establish normative percentiles for adolescents and young Spanish adults.

## 2. Materials and Methods

### 2.1. Study Design

We conducted a measurement properties evaluation study. To ensure a transparent, systematic, and reproducible assessment, we followed the COSMIN taxonomy and definitions of domains, measurement properties, and related concepts [20] when examining the psychometric performance of the AAC-Q in Spanish adolescents and young adults.

### 2.2. Measures

#### 2.2.1. Adolescents and Adults Coordination Questionnaire (AAC-Q)

The AAC-Q is a brief, self-report screening instrument developed to identify motor coordination difficulties in adolescents and young adults and to examine their impact on daily functioning. It assesses everyday tasks requiring motor coordination and organization, offering a context-based perspective of functional performance and allowing the detection of coordination difficulties that may continue into adolescence and adulthood [15].

The AAC-Q consists of 12 items, each rated on a 5-point Likert scale ranging from 1 (never [0%]) to 5 (always [100%]), yielding a total score from 12 to 60, with higher scores indicating greater motor coordination difficulties. The questionnaire includes items that reflect the individual’s experiences in daily life contexts such as self-care, handwriting, instrumental activities, leisure and social participation. Psychometric analyses reported by Tal-Saban et al. [15] demonstrated good internal consistency (α = 0.88), excellent test–retest reliability (r = 0.94, *p* < 0.001), and strong known-groups validity between adolescents with and without a clinical diagnosis of DCD (t(27) = 9.37; *p* < 0.001). The AAC-Q has been successfully cross-culturally adapted to Spanish young adults, showing excellent cross-cultural equivalence and good preliminary internal consistency (α = 0.74) [19]. The AAC-Q-ES may be requested from the first author.

#### 2.2.2. Comparator Instruments

Additionally, two more instruments were used for the measurement properties assessment: the ADC and the Flamingo Balance Test (FBT) from the EUROFIT battery [21].

The ADC is a brief self-report questionnaire developed to identify DCD in adults. It comprises 40 items distributed across three subscales: Subscale A assesses childhood motor difficulties (10 items), Subscale B explores how these difficulties affect current performance and participation (20 items), and Subscale C addresses the emotional and social consequences associated with them (10 items). Responses are rated on a four-point Likert scale (1 = never, 2 = sometimes, 3 = frequently, 4 = always), with a maximum total score of 120 points. Lower scores indicate better motor performance and functioning [14].

The ADC shows demonstrated strong internal consistency (Cronbach’s α = 0.95 (total scale); Subscale A: α = 0.91; Subscale B: α = 0.87; Subscale C: α = 0.90) [14]. The ADC served as the criterion measure to test the validity of the AACQ-ES in relation to difficulties in daily functional performance, corresponding to Criterion B of DCD.

The FBT, included in the EUROFIT fitness battery [21], is designed to measure static balance performance. As the ADC targets DCD-based daily performance issues during adulthood but not adolescence, the FBT test was used as a proxy measurement instrument for construct validity hypothesis testing in the adolescent sample. During the assessment, participants stand barefoot on a narrow beam (approximately 50 × 4 × 3 cm) and attempt to keep their balance on one leg, with the other leg lifted, for a total of one minute. The number of attempts required to maintain the posture is recorded as the test outcome.

The number of attempts recorded to complete the test allows for the classification of balance performance according to the European normative percentiles established in the EUROFIT battery [22]. A higher number of attempts indicates poorer balance control and consequently a lower percentile rank. According to Tomkinson et al. [22], performance levels are categorized based on these normative percentiles as follows: <P20: very low/poor; P20–P40: low; P40–P60: moderate; P60–P80: good; >P80: very good. Moreover, values ≤ P10 may be considered indicative of potential risk or functional deficit.

### 2.3. Sample Size Calculation

The estimation of the necessary sample size was guided by the methodological requirements of both exploratory (EFA) and confirmatory factor analyses (CFA). Factors such as the expected number of latent dimensions, communalities, factor loadings, and potential variations in the number of observed indicators were considered [16,23]. Since no prior reliable factorial structure had been proposed for the AAC-Q, a minimum sample of *n* ≥ 200 participants for each analysis was deemed appropriate to perform CFA testing one-, two-, or three-factor models, ensuring minimal estimation bias (α ≤ 0.05) and adequate statistical power (β ≥ 0.80). Additionally, a sample of *n* ≥ 19 was optimal for estimating test–retest reliability with minimal estimation bias and strongest statistical power (α ≤ 0.001; β ≥ 0.99) [15].

### 2.4. Procedure and Participants

Participants were recruited from several Universities and two high school centers in Spain following convenience sampling. Both university and high school students were invited to complete an online form that included the AAC-Q-ES, a sociodemographic questionnaire, and a question regarding the presence of any clinical condition or neurological disorder. All participants provided informed consent after receiving full information about the study aims and procedures, and that anonymity and confidentiality were guaranteed. University students also completed the Spanish version of the ADC, previously described, and high school students were administered the FBT, also previously described. Only typically developing students between 12 and 17 years, and between 18 and 35 years, who fully completed all measures, were included in the adolescent and young adult samples, respectively. The high school students completed a modified version of the AAC-Q-ES that did not include item 7 due to age- and cultural-based reasons. This decision was discussed with the main researchers of the European Spanish cross-cultural adaptation of the AAC-Q (L.D.-L. and R.M-M.), as item 7 described several tasks that Spanish adolescents do not engage in, such as driving a car or using a road map (i.e., ‘I have difficulty orientating in space like: getting lost easily, difficulty learning how to get to a new place, difficulty recognizing familiar driving routes, problems using a road map, difficulty finding your car in a car park, difficulty exiting a mall where I entered’). A consecutive sampling strategy was employed in the adolescent subsample, following age-representativeness criteria to achieve comparable group sizes of younger (12–14 years) and older (15–17 years) adolescents.

A total of 200 adolescents and 600 young adults who met the inclusion criteria were included in the study. The young adult sample was randomly divided into two separate samples to conduct EFA and CFA separately but was analyzed as a single sample during criterion validity testing and percentile determination. Age and sex distribution of both samples are displayed in Table A1.

A separate group of participants was recruited with the specific aim of assessing the test–retest reliability of the AAC-Q in Spanish young adults. This sub-sample included 20 undergraduate students enrolled in the Occupational Therapy program at the University of Murcia (southeastern Spain) who reported no prior diagnosis of neurodevelopmental disorders or disabilities. These students completed the AAC-Q-ES a second time three weeks after the initial administration to test test–retest reliability.

### 2.5. Analysis

The EFA (N = 300 young adults) was conducted with a minimum residual extraction method and an oblimin rotation with eigenvalues > 1 to identify the underlying structure of the ADC in the Spanish population. The items that loaded between 0.2–0.3 were revised and discussed by the first three authors on a one-to-one basis. The decision to delete or retain those items was made on the basis of content validity. A CFA was then calculated to confirm the produced latent structure (N = 200 adolescents + 300 young adults) using a diagonally weighted least squares (DWLS) estimation method. The structure fit was tested with the following indices and criteria: a Chi square/degrees of freedom (χ^2^/df) ratio ≤ 0.5 (preferred over the χ^2^ *p*-value due to its sensitivity to sample size), a Root Mean Square Error of Approximation (RMSEA) ≤ 0.08, a Goodness of Fit Index (GFI) ≥ 0.90, a Comparative Fit Index (CFI) ≥ 0.90, and a Tucker–Lewis Index (TLI) ≥ 0.90 [20]. This structure was then compared against the original unifactorial AAC-Q structure [15]. Internal consistency and test–retest reliability were tested using Cronbach’s alpha and intraclass correlation coefficients (respectively), with values ≥ 0.70 indicating good reliability. Following COSMIN recommendations, two predefined hypotheses were proposed to test construct and criterion validity of the AAC-Q in Spanish adolescents and young adults, expecting (a) a moderate (r ≥ 0.300) correlation between the AAC-Q total score and the percentile scores on the flamingo test for balance in adolescents; (b) a significant difference on the AAC-Q total score between those adolescents at functional risk (FBT ≤ 20th percentile) and those with typical development (FBT > 20th percentile), and; (c) a strong (r ≥ 0.600) correlation between the AAC-Q and the ADC total scores in young adults.

Finally, the 85th and 95th percentiles were determined for the AAC-Q-ES total score in Spanish adolescents and young adults. This approach follows international recommendations for the operationalization of diagnostic criteria for DCD [2,13], which advocate using the lowest 5th and 15th percentiles—or their corresponding 85th and 95th percentiles, as applied in the present study—from population-based distributions to determine cut-off points indicative of “substantially below expected motor competence” (criterion A) and “significant and persistent interference with age-appropriate daily living activities” (criterion B).

To establish sex- and age-specific cut-off values, linear regression analyses were performed separately for the adolescent and young adult samples to examine the effects of sex and age on AAC-Q scores (Table A2). In the adolescent sample, age group was shown to have a greater effect on total score than sex; therefore, age group-specific percentiles were calculated. In contrast, in the young adult sample, sex—but not age group (18–25 years vs. 26–35 years)—showed a significant effect, and sex-specific percentiles were consequently calculated.

## 3. Results

### 3.1. Structural Validity and Reliability

The EFA revealed a three-factor solution, with all items loading ≥ 0.3 except for item 7, which was the item already omitted for adolescents and loaded 0.258 on factor 1 for young adults. Thus, the 11-item and 12-item structures were compared in both samples, which yielded similar excellent results regarding fit adjustment for both adolescents and young adults (Table 1 and Table 2). Both three-factor models demonstrated a superior fit to the data compared with the original single-factor model (Table A3). Following conceptually and theoretically driven criteria, a decision was made to retain item 7 for young adults.

The AAC-Q-ES demonstrated good internal consistency for both adolescents (α = 0.83) and young adults (α = 0.76), as well as an excellent test–retest reliability (ICC = 0.901, *p* < 0.001).

### 3.2. Criterion Validity and Hypotheses Testing

All three predefined hypotheses regarding construct and criterion validity for the AAC-Q in Spanish adolescents and young adults were confirmed (Table 3 and Table 4).

### 3.3. Percentile Bands

Based on the age group- and sex-specific differences found on the multivariate analyses, specific percentile bands were calculated for Spanish adolescents and young adults (Table A3). Following current recommendations, the AAC-Q age group- and sex-specific 85th percentile on the total score should be used to operationalize criterion B for DCD in Spanish adolescents and young adults, respectively.

## 4. Discussion

This study provides robust evidence supporting the psychometric soundness of the European Spanish version of the AAC-Q for adolescent and young adult populations. The instrument demonstrated excellent internal consistency across both groups, high test–retest reliability in young adults, and very strong criterion validity with the ADC in young adults, as well as moderate correlations with the FBT in adolescents, as it would be expected. Furthermore, this is the first study to comprehensively examine the structural validity of the instrument, revealing a three-factor structure consistent with theoretical and experimental research. Overall, the findings support the AAC-Q-ES as a valid, reliable, and conceptually coherent tool for operationalizing Criterion B of DCD among Spanish adolescents and young adults.

The structural validity model of the AAC-Q-ES revealed a three-factor structure with excellent fit indices in both adolescent and adult populations, for the 11- and 12-item versions, respectively. These values exceed conventional thresholds and outperform those reported in previous adaptations of reference instruments, such as the ADC [24,25], commonly used for the assessment of DCD in adults. Comparisons with other adaptations or tools targeting adolescent populations were not possible, as the only available evidence to date stems from the brief mention by Tal-Saban et al. [15] in the original AAC-Q for adolescents and adults, which described a unifactorial structure explaining over 50% of the variance without providing detailed statistical data or confirmatory analyses to support it. Moreover, model fit testing indicated that the original single-factor model provided an inferior fit to the data compared with the three-factor model, suggesting that this three-factor solution reflects a more complex and multifactorial clinical manifestation of daily performance in youth with DCD.

Beyond its statistical robustness, the factorial solution obtained for the AAC-Q-ES reflects the main domains of functional difficulties described in the literature on DCD among adolescents and adults [26,27,28]. These domains encompass (1) manual performance skills, involving bimanual coordination and fine motor precision; (2) planning, organization, and task execution, related to time management and action sequencing; and (3) global movement patterns, associated with postural control and coordination in physical and sports activities. This structure demonstrates strong conceptual coherence with current theoretical frameworks of DCD, highlighting the transition from predominantly motor difficulties in childhood to more complex limitations in planning and executive control during adolescence and adulthood [2,11,29]. The AAC-Q-ES effectively reflects this developmental progression, integrating motor and processing dimensions that comprehensively represent the manifestations of the disorder across the lifespan.

The internal consistency of the AAC-Q-ES met the recommended criteria for both adolescents and young adults, supporting the conceptual coherence of the items within each factor. Test–retest reliability demonstrated excellent temporal stability, confirming the instrument’s reproducibility. This analysis was conducted in young adults using a three-week interval, following the procedure described in the original design study [15]. Although replication in adolescents was not possible due to logistical and ethical constraints, the results provide strong evidence of precision and stability. The α and ICC values are consistent with those reported in the original Israeli version of the AAC-Q [15] and slightly lower than those obtained for the ADC, both in its original version [14] and in subsequent adaptations [24,25,30].

Regarding criterion validity, the results revealed a very high correlation between the AAC-Q and the ADC in the adult population, supporting the equivalence between both measures and their utility for assessing daily performance difficulties associated with DCD. Both the ADC and the AAC-Q aim to assess daily performance difficulties in young adults to operationalize Criterion B, both in research and clinical settings. Unlike the ADC, which is considerably longer, the AAC-Q provides a brief, efficient, and sensitive alternative, facilitating its application in both clinical and research settings. Among adolescents, criterion validity could not be assessed due to the lack of an equivalent tool addressing criterion B for this developmental stage. Nevertheless, construct validity was supported through hypothesis testing, showing a moderate and significant negative correlation between the AAC-Q and the FBT, and significantly higher AAC-Q scores among adolescents with balance performance issues. These findings support the expected relationship between poorer motor performance and greater daily life difficulties, confirming the discriminative ability of the questionnaire.

Finally, the differences observed in AAC-Q-ES scores by age group and sex provide valuable insights for interpreting results and defining normative cut-offs. Among adolescents, significant variations emerged across age groups, with higher scores observed in the 15–17-year group. This pattern likely reflects the increased demands for planning, autonomy, and time management characteristics of late adolescence [2,31,32]. Conversely, the trend among adults was reversed: while no age-related effects were detected, women scored higher than men. This finding may be associated with a greater involvement in domestic and organizational responsibilities, which heightens perceived functional demands in daily life [31,33,34]. Based on these findings, age- and sex-specific percentile bands were established. Following current recommendations [2,13], the age- or sex-specific 85th percentile is proposed as the optimal cut-off for the operationalization of criterion B of DCD in Spanish adolescents and young adults.

This study presents several limitations that should be acknowledged. First, it was not possible to assess test–retest reliability or criterion validity in adolescents, due to ethical and logistical constraints in accessing minors and the absence of an equivalent reference instrument for this population. In addition, the use of convenience sampling may represent a potential source of bias, and the absence of a clinically diagnosed DCD sample limits the assessment of known-groups validity. Nevertheless, the results obtained provide a strong foundation for future research on the psychometric evaluation of daily functioning measures in adolescents and young adults with DCD. Importantly, these results may also assist clinicians and researchers in identifying individuals at risk of DCD in Spain, thus contributing to reducing some of these limitations. Future studies should aim to develop and validate criterion-referenced tools specifically designed for adolescents with DCD, as well as to conduct longitudinal and clinical studies that enable estimation of the sensitivity to change, minimal detectable change, and clinically meaningful differences of the AAC-Q-ES. Despite these limitations, the present work represents a pioneering contribution, providing the first empirical evidence of structural validity, reliability, and construct validity of the AAC-Q in adolescent and young adult populations. These findings support its applicability in both clinical and research settings, facilitating a more comprehensive assessment and understanding of functional difficulties associated with DCD across developmental stages.

## 5. Conclusions

The European Spanish version of the AAC-Q demonstrated strong psychometric robustness and conceptual coherence, confirming its suitability for assessing Criterion B of DCD in Spanish adolescents and young adults. This study provides the first comprehensive evidence of its structural validity, revealing a stable three-factor structure consistent with current theoretical models of DCD, alongside excellent internal consistency and temporal stability. Moreover, the AAC-Q-ES showed very high criterion validity with the ADC in adults and acceptable construct validity in adolescents, supporting its discriminative capacity across developmental stages. The establishment of age- and sex-specific percentile bands further enhances its interpretability and clinical utility.

Overall, the AAC-Q-ES stands out as a valid, reliable, and practical instrument for identifying and understanding functional difficulties associated with DCD from adolescence through early adulthood.

## Figures and Tables

**Table 1:** AFE factor loadings and CFA standardized coefficients for the 11-item and 12-item versions of the AAC-Q in Spanish adolescents and young adults.

	EFA Factor Loadings			CFA Standardized Estimates β		
				Adolescents	Young Adults	
Item	Factor 1	Factor 2	Factor 3	AAC-Q (11 Items)	AAC-Q (11 Items)	AAC-Q (12 Items)
Item 1		0.446		0.667	0.530	0.571
Item 2	0.461			0.710	0.643	0.606
Item 3		0.572		0.710	0.690	0.586
Item 4			0.360	0.708	0.530	0.648
Item 5		0.839		0.633	0.462	0.528
Item 6	0.487			0.706	0.590	0.413
Item 7	0.258			-	-	0.565
Item 8	0.586			0.789	0.712	0.709
Item 9	0.787			0.764	0.712	0.721
Item 10			0.945	0.740	0.678	0.693
Item 11	0.407			0.645	0.764	0.762
Item 12		0.342		0.809	0.786	0.788

Notes. 11-item version = without item 7.

**Table 2:** Comparison of the fit indices for 11-item and 12-item versions of the AAC-Q in Spanish adolescents and young adults.

Fit Indices	AAC-Q-ES Adolescents (11 Items)	AAC-Q-ES Young Adults (11 Items)	AAC-Q-ES Young Adults (12 Items)
X^2^/df	1.7	2.3	2.3
RMSEA (95% CI)	0.060 (0.035–0.0684)	0.065 (0.048–0.083)	0.067 (0.051–0.083)
GFI	0.99	0.98	0.98
CFI	0.98	0.95	0.95
TLI	0.98	0.93	0.93

Notes. 11-item version = without item 7; RMSEA = Root Mean Square Error of Approximation; GFI = goodness of fit index; CFI = comparative fit index; TLI = Tucker–Lewis index; 95% CI = 95% confidence interval.

**Table 3:** Correlations between the AAC-Q and the flamenco test (for construct validity testing in adolescents), and between the AAC-Q and the ADC (for criterion validity testing in young adults).

	FBT	ADC
AAC-Q-ES (adolescents)	−0.352 **	NA
AAC-Q-ES (young adults)	NA	0.972 ***

Notes. FBT = Flamingo Balance Test; ADC = Adult Developmental Co-ordination Disorders/Dyspraxia Checklist; NA = not applicable; ** = *p* < 0.01; *** = *p* < 0.001.

**Table 4:** Differences in AAC-Q-ES score between adolescents with or without developmental risk according to FBT.

	Developmental Risk (M [SD])	Typical Development (M [SD])	*p* Value
AAC-Q-ES total score	18.6 (7.0)	15.1 (4.2)	<0.001

Notes. FBT = Flamingo Balance Test; M = mean; SD = standard deviation.

## Data Availability

Restrictions apply to the datasets. The datasets presented in this article are not readily available because the data are part of an ongoing study. Requests to access the datasets should be directed to Prof. Laura Delgado-Lobete, who will individually review the request.

## Abbreviations

The following abbreviations are used in this manuscript:

DCD	Developmental coordination disorder
AAC-Q	Adolescents and Adults Coordination Questionnaire
ADC	Adult Developmental Co-ordination Disorders/Dyspraxia Checklist
FBT	Flamingo Balance Test
EFA	Exploratory factor analysis
CFA	Confirmatory factor analysis
X^2^/df	Chi square/degrees of freedom
RMSEA	Root Mean Square Error of Approximation
GFI	Goodness of fit index
CFI	Comparative fit index
TLI	Tucker–Lewis index
CI	Confidence interval
NA	Not applicable
M	Mean
SD	Standard deviation

## Appendix A

**Table A1:** Sample sex and age distribution.

	Adolescents	Young Adults (EFA)	Young Adults (CFA)
Age (M [SD])	14.7 (1.2)	22.8 (3.8)	23.0 (4.1)
12–14 yrs (%)	50%	-	-
15–17 yrs (%)	50%	-	-
18–25 yrs (%)	-	79%	76
26–35 yrs (%)	-	21%	24
Female (%)	49%	68%	62%

**Table A2:** Linear regression predicting AAC-Q scores from age and sex.

Predictor	β	t	*p* Value
Adolescents			
Sex (female vs. male)	0.147	2.098	0.037
Age group (12–14 yrs vs. 15–17 yrs)	−0.209	−2.978	0.003
Young adults			
Sex (female vs. male)	0.149	3.691	<0.001
Age group (18–25 yrs vs. 26–35 yrs)	0.006	0.143	0.887

**Table A3:** Fit indices for the original single-factor model proposed by Tal-Saban et al. [15] in Spanish young adults.

Fit Indices	AAC-Q-ES Young Adults (Original Model)
X^2^/df	3.7
RMSEA (95% CI)	0.095 (0.081–0.109)
GFI	0.97
CFI	0.89
TLI	0.86

Notes. RMSEA = Root Mean Square Error of Approximation; GFI = goodness of fit index; CFI = comparative fit index; TLI = Tucker–Lewis index; 95% CI = 95% confidence interval.

**Table A4:** Percentile bandings of AAC-Q for Spanish adolescents and young adults.

Percentile	AAC-Q-ES (Total Score)
Adolescents (12–17 yrs; 11-item version) 12–14 yrs	
85th	25
95th	32
15–17 yrs	
85th	22
95th	26
Young adults (18–35 yrs; 12-item version)	
Females	
85th	25
95th	30
Males	
85th	22
95th	26

Note. 11-item version = without item 7.
